# Risk prediction models for venous thromboembolism in stroke: a systematic review and meta-analysis

**DOI:** 10.3389/fcvm.2026.1788144

**Published:** 2026-04-23

**Authors:** Lina Fu, Chunyan Cui, Kairu Feng, Xinjie Li, Meng Wang, Zubair Muhammad, Nan Liu

**Affiliations:** 1 Institute of Environment and Health, South China Hospital, Medical School, Shenzhen University, Shenzhen, China; 2 Institute of Chronic Disease Risks Assessment, School of Nursing and Health, Henan University, Kaifeng, China

**Keywords:** meta-analysis, prediction model, stroke, systematic review, venous thromboembolism

## Abstract

**Objective:**

This study aimed to systematically evaluate risk prediction models for venous thromboembolism (VTE) in stroke patients and to provide a reference for future model development and clinical research.

**Methods:**

A systematic search was conducted across multiple databases, including PubMed, Embase, Web of Science, the Cochrane Library, China National Knowledge Infrastructure (CNKI), Wanfang Database, VIP, and SinoMed, to identify studies on VTE risk prediction models in stroke patients. Databases were searched from inception to September 1, 2025. Risk of bias and applicability of the prediction models were assessed using the PROBAST checklist. Meta-analyses were conducted to estimate pooled VTE incidence and the area under the curve (AUC) for model performance using Stata 17.0.

**Results:**

A total of 2,726 records were retrieved, and seven prediction models were included. Reported VTE incidence ranged from 9.8% to 38.9%, with AUC values between 0.781 and 0.978, indicating moderate to high apparent discriminative performance, however, this should be interpreted with caution in light of the high risk of bias and lack of external validation. None of the seven included models underwent independent external validation, and according to PROBAST, all included models were judged to be at high overall risk of bias. The pooled VTE incidence was 20.8% (95% CI: 14.7%–27.0%), and the pooled AUC across six models was 0.87 (95% CI: 0.81–0.93).

**Conclusion:**

Among the VTE risk prediction models for stroke patients included in this study, although some models demonstrated favorable predictive performance, all models were judged to be at high risk of bias according to PROBAST. Future research should prioritize the validation and refinement of existing models or the development of new models with more rigorous methodological design, in order to better support clinical decision-making for patients with stroke.

**Systematic Review Registration:**

https://www.crd.york.ac.uk/prospero/display_record.php?ID=CRD42024603132, PROSPERO CRD42024603132.

## 1 Introduction

Stroke is a rapidly developing focal or global brain dysfunction lasting more than 24 h or resulting in death (1–4). Stroke is one of the leading causes of death and long-term disability worldwide (5), particularly in low- and middle-income countries (6). There are approximately 10.3 million new cases of stroke worldwide each year (7). In China, stroke has become a major chronic non-communicable disease that poses a serious public health challenge. It is the leading cause of death and disability among adults and is characterized by high incidence, high disability, high mortality, high recurrence, and a substantial economic burden (8).

With continuous advances in stroke treatment technologies and significant improvements in the quality of in-hospital care, stroke-related mortality has declined (9). However, despite these advancements, stroke patients are still prone to secondary complications (10) instance, venous thromboembolism (VTE), including deep vein thrombosis (DVT) and pulmonary embolism (PE), is a frequent yet underdiagnosed complication of stroke characterized by insidious and non-specific symptoms (11). Stroke patients who experience VTE face a significantly increased risk of poor prognosis and death within three months (12). Beyond its impact on patient outcomes, VTE also imposes a considerable burden on healthcare systems, as it is frequently associated with hospitalization, prolonged length of stay, and increased healthcare resource utilization (13), early identification of high-risk patients and timely implementation of preventive interventions are crucial for reducing the incidence of VTE, improving clinical outcomes, and enhancing quality of life in stroke survivors.

Prediction models estimate the probability of the occurrence of a specific outcome by integrating multiple predictors (14). They are widely applied in clinical practice and public health decision-making (15). Prediction models enable clinicians to identify high-risk individuals early and to implement tailored preventive interventions according to model-based risk stratification, thereby improving clinical outcomes (16). In recent years, researchers have developed several VTE prediction models specifically for stroke patients (17–23). However, although several studies have explored VTE risk factors or individual prediction models in stroke patients, systematic comparative evaluations of these models in terms of modeling methods, predictive performance, and sample-related bias remain limited, particularly with respect to recently developed models and those derived from Chinese populations. Therefore, it remains unclear which model is more suitable for stroke patients. Therefore, this study aims to systematically evaluate VTE prediction models for stroke patients both in China and internationally, to provide a reference for future model development and clinical application.

## 2 Methods

### 2.1 Design

We reported this review according to the guidelines of the Preferred Reporting Items for Systematic Reviews and Meta-Analyses (PRISMA) (24). This study was registered prospectively in PROSPERO (CRD42024578643).

### 2.2 Retrieval strategy

The databases searched included PubMed, Embase, Web of Science, Cochrane Library, CNKI, Wanfang Database, VIP, and SinoMed. The search period spanned from the inception of each database to September 1, 2025, using the following keywords: “stroke”, “venous thromboembolism”, “risk prediction model”, “risk score”, “risk factors”, “predictors”, and “nomogram”, and “model”.

### 2.3 Eligibility criteria

The inclusion criteria for this study were developed according to the PICOTS (25) framework and detailed in Table 1. Studies that were limited to VTE subgroups, as well as reviews, case reports, and other informally published or duplicated studies, were excluded. Additionally, studies for which the full text was unavailable or for which the model details were incomplete were also excluded.

**Table 1:** Criteria for study inclusion in the systematic review.

PICOTS framework	Definition
Population	Patients aged ≥18 who meet the diagnostic criteria for stroke.
Intervention	Published risk prediction models for VTE in stroke patients incorporating at least two predictors.
Comparator	Not applicable.
Outcome	Outcomes focused specifically on VTE rather than its subgroups.
Timing	Timing involves predicting results based on the patient's baseline information, clinical scoring scales, and laboratory test results.
Setting	Settings focus on personalized prediction for stroke patients, facilitating early identification of high-risk VTE groups and enabling the implementation of targeted preventive measures.

### 2.4 Literature screening and data extraction

Two researchers trained in evidence-based medicine independently screened the literature and extracted data based on the inclusion and exclusion criteria. Disagreements were resolved through discussion with a third reviewer (26). The data collection table was developed based on the CHARMS checklist for critical appraisal and data extraction in systematic reviews of prediction modeling studies (27), and includes the following: (1) basic characteristics of the included studies (author, publication date, country, data source, and sample size), (2) establishment of the prediction model (modeling method, number of models, handling of missing data, treatment of continuous variables, number of predictors, and factors included), (3) performance and presentation of the prediction model (model performance, model validation, and model presentation), and (4) quality evaluation of the prediction model (research subjects, predictors, results, and statistical analysis).

### 2.5 Quality evaluation

Two researchers independently used the prediction model risk of bias assessment tool (PROBAST) (28) to assess the risk of bias and applicability of the included studies. Discrepancies in the evaluation results were resolved through discussion with the third party.

When multiple predictive models are developed using different methodologies within a single study, the model recommended by the authors as the best is prioritized for evaluation. If no model is explicitly defined, the one with the highest predictive performance is selected for assessment. The risk of bias assessment evaluates four key areas: research subjects, predictors, results, and statistical analysis, consisting of 20 questions in total. Each answer was as “yes/maybe”, “no/maybe not”, or “no information”. The evaluation results for each area were classified into three categories: low, high, or unclear risk of bias. If all four areas indicated low risk, the study is considered to have a low risk of bias. A high risk in any area, or a lack of external validation despite low risk in all areas, results in a high overall risk of bias. If any area has an unclear result, while the others showed a low risk of bias, the study is classified as having unclear overall risk of bias. The applicability evaluation focuses on three areas: research subjects, predictors, and results, and follows a process similar to the risk of bias evaluation.

### 2.6 Statistical analysis

Meta-analysis was performed using Stata 17.0 to evaluate the incidence of VTE in stroke patients and the area under the curve (AUC) of the prediction models. Pooling of AUC values was conducted to provide an overall summary of the discriminative performance reported in the included studies. The pooled AUC was interpreted as a descriptive rather than inferential estimate and was not intended for direct comparison between models. Heterogeneity was assessed using the *I*^2^ statistic and the Cochrane *Q* test. The *I*^2^ statistic was used to quantify heterogeneity, with values of 25%, 50%, and 75% representing low, moderate, and high heterogeneity, respectively. A fixed-effects or random-effects model was selected according to the degree of heterogeneity (29).

## 3 Results

### 3.1 Literature screening process and results

**Figure 1:**
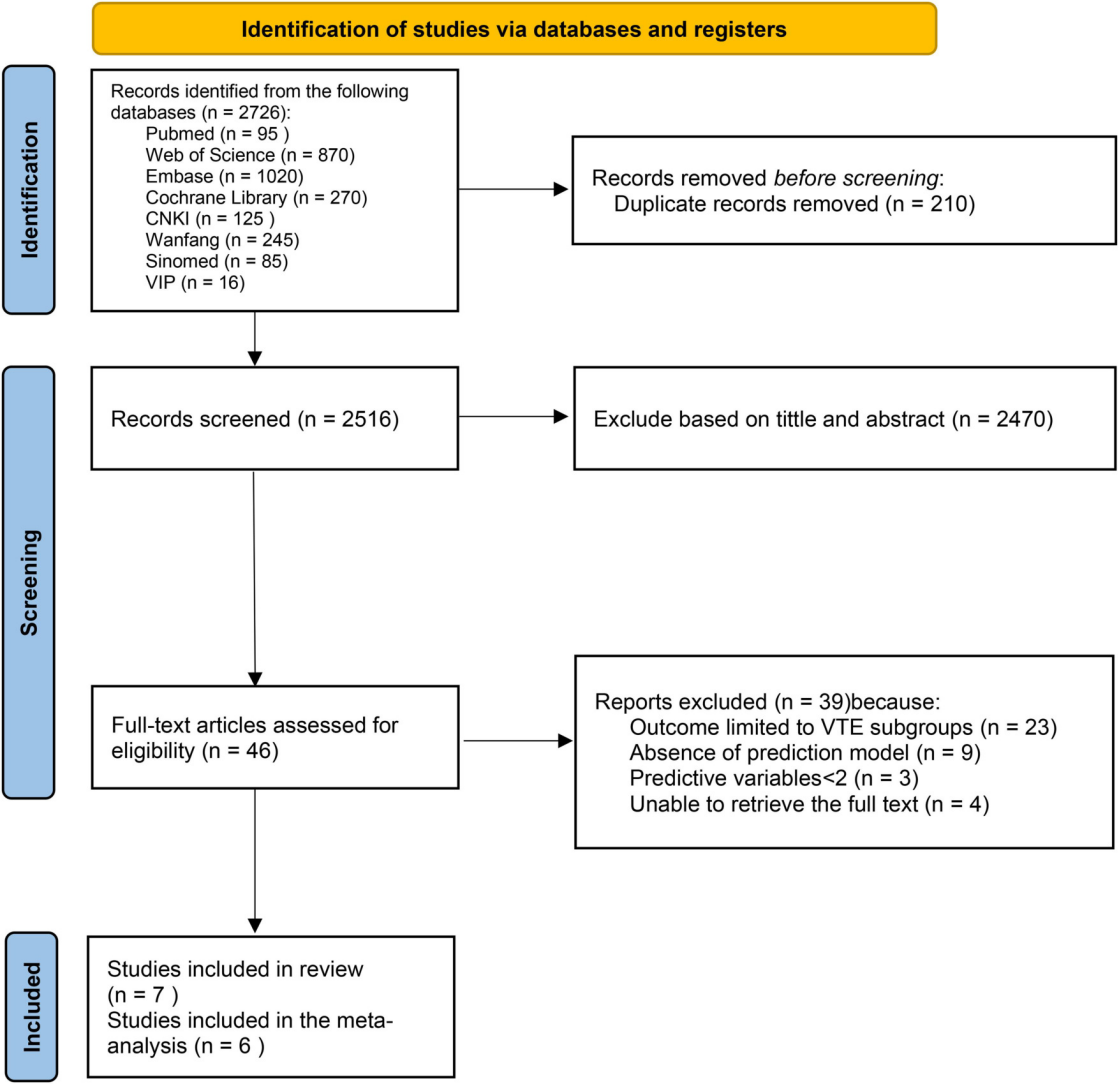
PRISMA flow diagram of the literature search and study selection process.

A search of the relevant databases initially identified 2,726 articles, of which 2,516 remained after removing 210 duplicates. These articles were then screened according to predefined inclusion and exclusion criteria. Initially, 2,470 articles were excluded following a review of the title and abstract, and 46 articles remained and were evaluated in full text. Of these, 23 articles focused exclusively on outcomes related to the VTE subgroup, 9 articles addressed risk factors without developing prediction models, 3 articles included fewer than two predictors, and 4 articles were inaccessible. Consequently, 39 articles were excluded. Finally, 7 articles were included in this study, a total of 7 studies were included (17–23). The literature screening process and results are depicted in Figure 1.

### 3.2 Basic characteristics of the included literature

The seven studies included in this analysis were published between 2022 and 2025, all using a retrospective design. Five of them are Chinese articles (17, 19, 20, 22, 23), and the other two are English articles (18, 21), respectively. One study was sourced from the MIMIC-IV database (18), while another utilized data from the Henan Stroke Cohort (19). We also found that five studies focused on stroke patients in general (17–20, 22), the other two studies specifically addressed ischemic stroke (23) and hemorrhagic stroke (21). The total sample size ranged from 185 to 675, with the number of VTE cases among stroke patients varying from 26 to 164, with an incidence rate between 9.8% and 38.9%. The basic characteristics of the included studies were summarized in Table 2.

**Table 2:** Overview and characteristics of the included studies.

Author (year)	Participants	Data source	Cases/sample size (%)
Su Wei (2025)^a^	Stroke Patients	The neurology department of a general hospital (2022–2024)	72/185 (38.9%)
Folin Lan (2024)	Stroke Patients	MIMIC-IV Database (2008–2019)	26/266 (9.8%)
Wang Rongrong^a^ (2023)	Stroke Patients	Henan Stroke Cohort (2019–2021)	164/675 (24.3%)
Hu Yonghuan^a^ (2023)	Stroke patients aged ≥ 18 years and hospitalized for ≥ 48 h	HIS system of a tertiary hospital (2021–2022)	49/409 (11.98%)
Liu Jie^a^ (2022)	Acute stroke patients aged ≥ 18 and hospitalized within 24 h after onset.	EMR of one hospital (2018–2020)	38/215 (17.67%)
Cao Hua^a^ (2022)	Acute ischemic stroke patients aged ≥ 18 years and diagnosed within 72 h after onset	The neurology department of a general hospital (2019–2020)	55/238 (23.1%)
Liu Shucheng (2022)	Primary ICH patients aged ≥ 18 years who undergo screening within 24 h of onset	Neurosurgery intensive care unit of a certain hospital (2019–2021)	83/369 (22.5%)

^a^ This study was published in Chinese.

### 3.3 Construction of prediction model

A total of nine VTE risk prediction models for stroke patients were developed across seven studies. One study developed two prediction models using logistic regression and random forest (19), while the remaining six studies used logistic regression to develop their models (17, 18, 20–23). In addressing missing data, the studies employed varying approaches. One study utilized the random forest method for datasets with a missing rate below 20% (18), while another applied multiple imputation methods (19). Four studies employed complete case analysis (17, 20, 22, 23), and one study did not specify whether missing data were present or how they were handled (21). Regarding the treatment of continuous variables, methodological differences were also apparent. Four studies preserved the continuous nature of these variables (17, 18, 21, 23), whereas the remaining three studies converted all continuous variables into categorical variables (19, 20, 22). A comprehensive summary of the prediction models is presented in Table 3.

**Table 3:** Basic information of the prediction models.

Author (year)	Model development method	Model numbers	Missing data handling	Continuous variables processing method	Final predictors
Su Wei (2025)	LR	1	Complete-case analysis	Maintain continuity	Intracranial hemorrhage volume, GCS score, FIB, CRP, D-dimer
Folin Lan (2024)	LR	1	Exclude if missing >20%, otherwise RF imputation	Maintain continuity	Log-formed D-dimer, PTT, Lung infection, MCH
Wang Rongrong (2023)	LR, RF	2	Multiple imputation	Categorical variables	Age, ADL, Muscle strength, UA, Length of stay, Fib, TC, D-dimer,
Hu Yonghuan (2023)	LR	1	Complete-case analysis	Categorical variables	Age, History of VTE, ADL, D-dimer
Liu Jie (2022)	LR	1	Complete-case analysis	Categorical variables	History of VTE, D-dimer, Recent surgical history, Bed rest time, Central venous catheterization, Prophylactic anticoagulation
Cao Hua (2022)	LR	1	Complete-case analysis	Maintain continuity	CRP, D-dimer
Liu Shucheng (2022)	LR	1	NA	Maintain continuity	GCS score, NIHSS score, D-dimer

LR, logistic regression, RF, random forest, NA, not reported, PTT, partial thromboplastin time, MCH, mean corpuscular hemoglobin, ADL, activity of daily living, UA, uric acid, FIb, fibrinogen, TC, total cholesterol, CRP, C-reactive protein, GCS, Glasgow Coma Scale, NIHSS, National Institute of Health stroke scale.

### 3.4 Performance of prediction models

This study analyzed AUC values for the included models, ranging from 0.78 to 0.98, with sensitivity ranging from 70% to 94.4%, and specificity ranging from 67.35% to 91.6%. All seven studies reported both discrimination and calibration metrics. Among them, five studies assessed calibration using the Hosmer–Lemeshow test, and five studies reported calibration curves. However, more informative measures of calibration, such as calibration slope, intercept, or Brier score, were not reported in any of the included studies. In terms of model validation, one study did not conduct internal validation (22). The other six studies used internal validation using random split validation, k-fold cross-validation, bootstrap repeated sampling (17–21, 23). None of the seven studies performed external validation. For model presentation, six studies primarily utilized nomogram score analysis to illustrate their predictions (17–21, 23), while one study proposed a risk score formula (22). A detailed summary of the performance and presentation formats of the prediction models is provided in Table 4.

**Table 4:** Performance and presentation formats of the prediction models.

Author (year)	Model performance	Calibration method	Validation methodology	Model presentation
Su Wei (2025)	A: 0.98 (0.94–0.99)	CC	Random split	Nomogram
	B: 0.96 (0.89–0.99)			
Folin Lan (2024)	A: 0.88	H-L test, CC	Random split	Nomogram
	B: 0.88			
Wang Rongrong (2023)	A: 0.92 (0.90–0.95)	H-L test	Random split 5-fold cross	Nomogram
	B: 0.90 (0.85–0.95)			
Hu Yonghuan (2023)	A: 0.78 (0.71–0.85)	H-L test, CC	Bootstrap	Nomogram
	B: 0.74 (0.67–0.82)			
Liu Jie (2022)	A: 0.81 (0.73–0.89)	H-L test, CC	None	Risk score formula
Cao Hua (2022)	A: 0.90 (0.85–0.96)	CC	Bootstrap	Nomogram
Liu Shucheng (2022)	A: 0.79 (0.74–0.85)	H-L test, CC	Bootstrap	Nomogram

AUC, Area under the curve (0.5–0.7 as poor discrimination, 0.7–0.8 as moderate discrimination, 0.8–0.9 as good discrimination, and 0.9–1.0 as excellent discrimination), A, development cohort, B, validation cohort, H-L test, Hosmer-Lemeshow Goodness of fit test, CC, calibration curve. The study by Folin Lan (2024) was excluded from the AUC meta-analysis because confidence intervals were not reported.

### 3.5 Literature quality evaluation

#### 3.5.1 Risk assessment of bias

All seven studies were assessed with a high overall risk of bias. Primarily, due to the retrospective nature of the studies, it may introduce recall bias. Additionally, the data in these studies were not originally collected with the intent of developing or validating prediction models. Consequently, key predictors related to VTE in stroke patients may not have been fully documented in the cases. In the area of predictors, five studies were assessed to have a low risk of bias (17, 20–23), whereas two were rated as an unclear risk of bias (18, 19). This uncertainty arose primarily from the lack of reported quality control during the collection and evaluation of predictors, leading to inconsistencies in both timing and method of data acquisition. Furthermore, the absence of standardized training or guidelines for raters may have further contributed to variability and potential bias. In the assessment of results, six studies were rated as having a low risk of bias. However, one study (18) failed to specify the criteria used to define outcomes, raising concerns about the reliability and reproducibility of its findings.

In the area of statistical analysis, all seven studies exhibited a high risk of bias. Importantly, the high overall risk of bias observed in the included studies was primarily driven by deficiencies in the analysis domain, rather than the absence of external validation alone. Several methodological shortcomings were identified, and the main reasons included that six studies were unable to satisfy the recommended number of events per variable (EPV) ≥20 for each independent variable (17, 18, 20–23), three studies transformed all continuous variables into categorical variables (19, 20, 22), one study did not disclose the method used to handle missing data (21), and six studies relied on univariate analysis to screen predictors. Additionally, two studies did not address the key issues such as overfitting, underfitting, or optimal fitting during their modeling prediction (18, 22), and two studies failed to report the coefficients of predictors within their models (18, 21). The PROBAST assessment results were summarized in Table 5.

**Table 5:** PROBAST results for the included studies.

Author (year)	Study type	ROB				Applicability			Overall	
		Participants	Predictors	Outcome	Analysis	Participants	Predictors	Outcome	Risk of Bias	Applicability
Su Wei (2025)	B	−	+	+	−	+	+	+	−	+
Folin Lan (2024)	B	−	?	?	−	+	+	+	−	+
Wang Rongrong (2023)	B	−	?	+	−	+	+	+	−	+
Hu Yonghuan (2023)	B	−	+	+	−	+	+	+	−	+
Liu Jie (2022)	A	−	+	+	−	+	+	+	−	+
Cao Hua (2022)	B	−	+	+	−	−	+	+	−	−
Liu Shucheng (2022)	B	−	+	+	−	−	+	+	−	−

PROBAST, Prediction model Risk Of Bias Assessment Tool, ROB, risk of bias.

A, indicates “development only”; B, indicates “development and validation in the same publication”; +, indicates low ROB/low concern regarding applicability; −, indicates high ROB/high concern regarding application;?, indicates unclear ROB/unclear concern regarding applicability.

#### 3.5.2 Applicability risk assessment

Five studies demonstrated a low risk of applicability concerns (17–20, 22), whereas two studies (21, 23) were classified as having a high risk of applicability. The high risk in these studies stemmed from their limited focus. The two studies focused on hemorrhagic stroke (21) and acute ischemic stroke (23), respectively. Consequently, the present predictive models developed in these studies are lacking in generalizability to the broader population of stroke patients.

### 3.6 Meta-analysis results

A meta-analysis was conducted to estimate the incidence of VTE in stroke patients across the seven included studies. Substantial heterogeneity was observed among the studies (*I*^2^ = 93.4%, Cochran's *Q* = 84.91, df = 6, *P* < 0.001).

**Figure 2:**
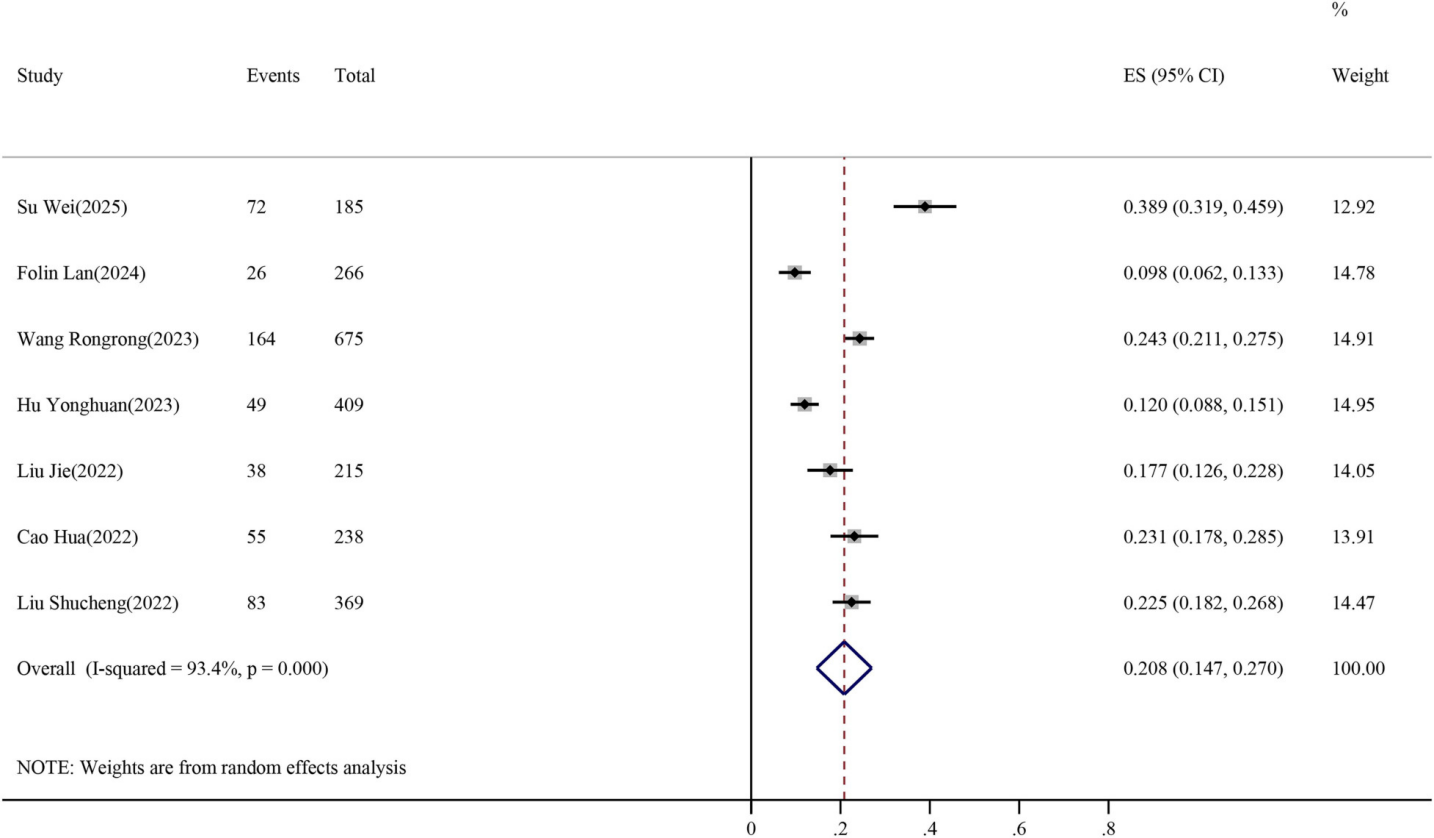
Forest plot of pooled VTE incidence in stroke patients.

Sensitivity analysis using a leave-one-out approach demonstrated that exclusion of any single study did not materially change the pooled estimate, indicating the robustness of the overall result. However, heterogeneity remained consistently high across all iterations. Due to the limited number of included studies (*n* = 7), formal subgroup analyses were not performed, as this would result in an insufficient number of studies per subgroup and reduce statistical reliability. Given the substantial between-study variability, a random-effects model was applied to account for heterogeneity. The pooled incidence of VTE in stroke patients was 20.8% (95% CI: 14.7%–27.0%), as shown in Figure 2. Forest plot of pooled VTE incidence in stroke patients. The horizontal axis represents the proportion of VTE incidence. Weights were assigned using the DerSimonian–Laird random-effects model.

**Figure 3:**
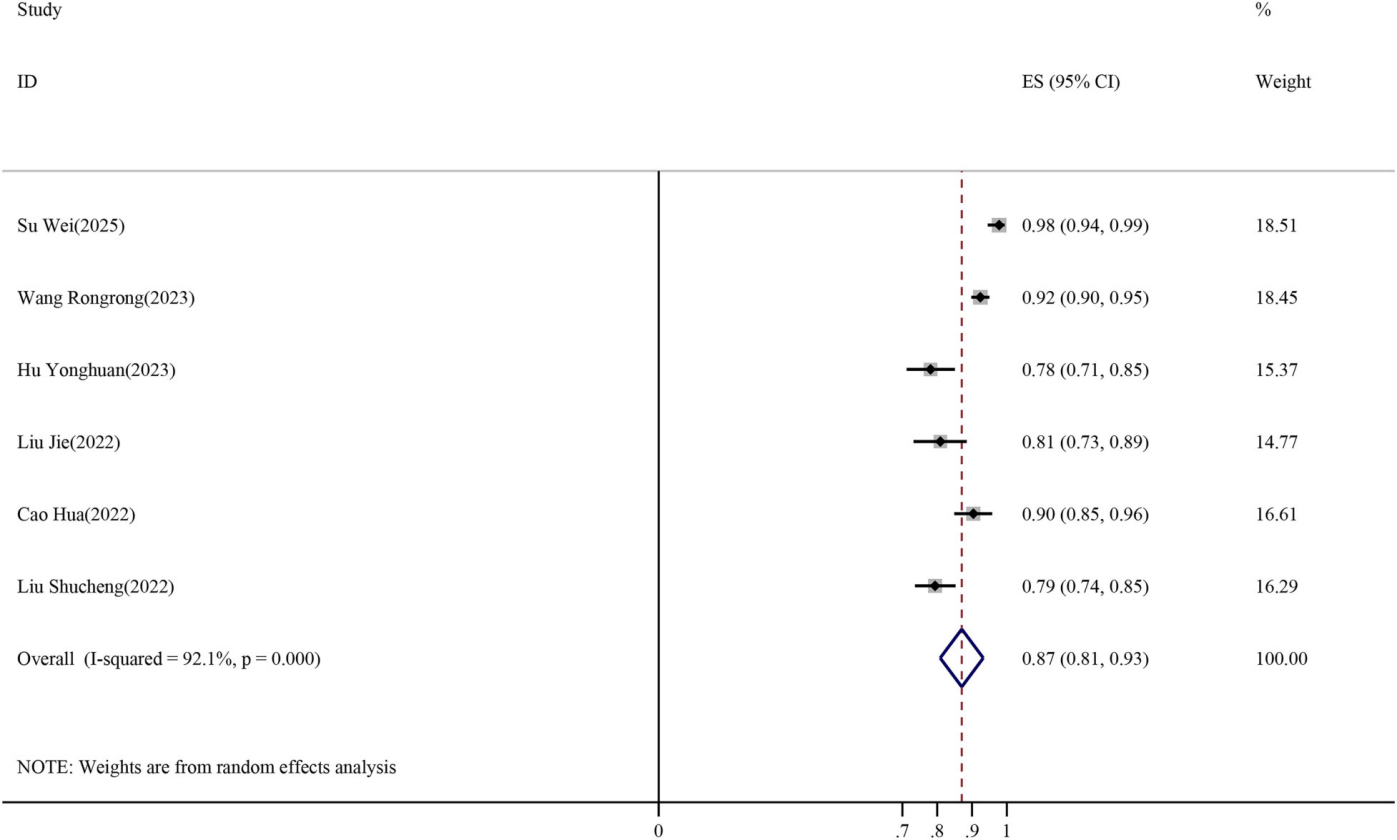
Forest plot of pooled AUC values for VTE risk prediction models in stroke patients.

Subsequently, a meta-analysis was conducted to summarize the AUC values reported for the prediction models. One model was excluded due to the absence of a reported 95% confidence interval (18), and six models were included in the analysis (17, 19–23). Among these, one study developed two models based on the same dataset, and one model was selected to avoid duplication (19). Substantial heterogeneity was observed (*I*^2^ = 92.1%, Cochran's *Q* = 58.29, df = 5, *P* < 0.001). Given the substantial between-study variability, a random-effects model was used, yielding a pooled AUC of 0.87 (95% CI: 0.81–0.93), as shown in Figure 3. Forest plot of pooled AUC values for VTE risk prediction models in stroke patients. The horizontal axis represents AUC values. Weights were assigned using the DerSimonian–Laird random-effects model.

## 4 Discussion

### 4.1 Main findings

Risk prediction models can help stratify VTE risk in stroke patients, enabling clinicians to plan targeted preventive interventions according to predicted risk. This systematic review identified seven VTE risk prediction models for stroke, with reported AUCs ranging from 0.781 to 0.978. Based on the reported AUC values, the included models appeared to show moderate to high discriminative performance. The pooled AUC of the included models was 0.87 (95% CI: 0.81–0.93), however, given the substantial heterogeneity across studies, this estimate should be interpreted as a descriptive summary of reported discriminative performance rather than as evidence of directly comparable model performance. According to PROBAST, all included studies were judged to have a high overall risk of bias, and two models were also judged to have high concern regarding applicability. Accordingly, these estimates should be interpreted with caution, as methodological limitations—including retrospective study designs, limited numbers of outcome events, suboptimal handling of missing data, and the absence of independent external validation—are likely to have resulted in overfitting and optimistic bias, thereby inflating the reported model performance. In addition, calibration assessment in the included studies was limited. Most studies relied on the Hosmer–Lemeshow test, which has known limitations, including sensitivity to sample size and limited ability to evaluate agreement between predicted and observed risks. Although several studies reported calibration curves, more informative quantitative measures, such as calibration slope, intercept, or Brier score, were not reported. Importantly, good discrimination alone is insufficient for clinical application. In the context of VTE prevention after stroke, inaccurate risk stratification may lead to inappropriate preventive strategies, such as unnecessary anticoagulation, thereby increasing the risk of bleeding complications (30). Therefore, future studies should incorporate comprehensive evaluation of both calibration and clinical utility, including methods such as decision curve analysis, to better support clinical decision-making.

In addition to model performance, substantial heterogeneity was observed in the pooled incidence of VTE across studies. Due to the limited number of included studies, formal subgroup or meta-regression analyses were not feasible. Therefore, potential sources of heterogeneity were explored qualitatively. This heterogeneity is likely attributable to both clinical and methodological differences across studies. On the one hand, the study populations were not uniform. Some studies enrolled broadly defined stroke patients from neurology wards or hospital information systems, whereas others focused on more selected subgroups, such as patients with acute ischemic stroke within a specific time window after onset or patients with primary intracerebral hemorrhage treated in neurosurgical intensive care units. On the other hand, data sources varied considerably, including single-center electronic medical records, hospital information systems, regional stroke cohorts, and large clinical databases. Differences in inclusion time criteria (e.g., within 24, 48, or 72 h after stroke onset) may have further influenced sample composition and outcome ascertainment.

Furthermore, although VTE was consistently defined as the outcome, diagnostic strategies and timing of assessment were not uniform across studies. These variations may have affected VTE detection and partly explain the observed variability in incidence estimates. Taken together, these findings suggest that the substantial heterogeneity is likely driven by genuine clinical and methodological diversity rather than random variation alone.

Given the high risk of bias and the lack of independent external validation, prediction models for VTE in stroke patients remain at a developmental stage. The direct application of existing models in routine clinical practice, therefore, remains controversial, and further external validation and/or recalibration is essential. Despite these limitations, prediction models may still have potential value in specific clinical scenarios. In current stroke care pathways, VTE prophylaxis is largely guided by general clinical recommendations, however, individualized risk stratification remains limited. Well-validated and well-calibrated prediction models could help identify patients at particularly high or low risk of VTE, especially in situations where the decision to initiate or intensify prophylaxis is uncertain. At the same time, premature implementation of inadequately validated models may lead to inappropriate clinical decisions. In particular, overestimation of VTE risk may result in unnecessary anticoagulation, thereby increasing the risk of bleeding complications. Therefore, careful validation and evaluation of clinical utility are essential before such models can be safely integrated into routine practice. In addition, evolving clinical contexts and emerging risk factors may further influence the epidemiology and management of VTE (31), underscoring the need for continuous updating and validation of prediction models in changing clinical environments.

### 4.2 Sources of bias in prediction models

Prediction models developed using retrospective designs may contain incomplete data or recording bias, and potential predictors may be excluded because of missing information (32), which can contribute to biased coefficient estimates and overfitting. Therefore, prospective cohort studies or nested case-control designs are preferable for prediction model development to mitigate these issues. Such study designs enhance data integrity and consistency, thereby reducing the risk of data bias (33). Notably, two studies failed to specify whether predictors were collected and evaluated independently of outcome data (18, 19), raising concerns about potential confounding factors. Additionally, inconsistencies in the measurement methods, processes, and timing of predictor assessments across different medical institutions also remained unaddressed. Thus, to ensure the reliability and accuracy of prediction models, standardized procedures for the measurement and evaluation of predictors should be established (4). In multi-center studies, uniform training for data collectors, the application of blinding methods during data collection, and rigorous quality control measures are essential to ensure data integrity and reliability (34).

Currently, most studies still rely on the widely accepted “10 EPV” principle to calculate sample size. However, this minimum sample size rule of thumb is overly simplistic and fails to account for the complexity of modern prediction modeling (35, 36), which may increase the risk of overfitting and lead to overly optimistic performance estimates. Adequate sample size determination for a prediction model requires careful and detailed consideration of multiple factors beyond the number of candidate predictors, including the total sample size, proportion of outcomes (incidence) within the study population, and the anticipated predictive performance of the model (35, 37). Emerging methodologies, including simulation-based approaches and machine learning–informed frameworks, have been proposed to support more precise sample size estimation (38). Additionally, advanced data integration techniques can effectively expand the sample size, thereby improving the reliability and generalizability of predictive models.

Several of the included studies failed to specify their approach to handling missing data (21), while others relied solely on complete datasets for analysis (17, 20, 22, 23). Improper management of missing values can significantly compromise model development, often resulting in overfitting or biased coefficient estimates and consequently leading to overly optimistic assessments of model performance. Complete case analysis, although simple to implement, excludes a substantial portion of the dataset, reducing statistical power and generalizability. Similarly, simple single-imputation approaches, while convenient, may increase variable homogeneity and adversely affect model fitting by oversimplifying data relationships. In contrast, single imputation leverages the correlation between variables but introduces an element of randomness. Multiple imputation, as a more robust approach, imputes missing data *n* times to generate *n* complete data sets, followed by a comprehensive statistical analysis across these data sets to derive the imputation results (39). To ensure data integrity and maximize the utility of valuable information, researchers should carefully select an appropriate imputation strategy that aligns with the specific characteristics of the data. Additionally, reliance on univariate screening methods for predictor selection may introduce estimation bias and increase the risk of overfitting, thereby weakening the apparent predictive performance. Statistical analysis methods such as Lasso regression and ridge regression, combined with clinical expertise, offer robust alternatives to mitigate the risk of model overfitting and enhance predictor selection accuracy to ensure more reliable and interpretable models (40).

Some studies directly transformed continuous variables into categorical ones, grouping them into two or more categories without clearly specifying the criteria for these divisions (19, 20, 22), which may lead to information loss and increase the risk of overfitting. This approach can result in a loss of valuable information and a reduction in statistical power. Although categorical variables may be easier to interpret, continuous variables retain a broader range of values, which can improve the precision of models (41).

Evidence suggests that predictive models retaining continuous variables often outperform those using categorical representations, particularly when critical thresholds are arbitrarily set (42). For internal model validation, two studies employed a random split method (7:3) (17, 18), which may result in inefficient use of data and increase the risk of overly optimistic performance estimates. As a result, this approach may lead to inefficient use of data and reduced statistical power (43). More robust alternatives, such as K-fold cross-validation and bootstrapping, are recommended to enhance model reliability (44). In multicenter studies, an internal-external cross-validation approach provided an effective strategy to assess model performance across diverse settings (45). Additionally, external validation is essential for evaluating the portability and generalizability of a model (46). However, none of the seven included studies conducted external validation, raising concerns about the applicability of their findings. Future studies should ensure the external validation is performed independently of model development to maintain objectivity and rigor without involvement in the initial modeling process (4).

### 4.3 Analysis of predictors

This study included seven VTE risk prediction models, encompassing 19 distinct predictors, which reflects the diversity of variable selection in existing models. In terms of frequency, D-dimer (7 occurrences) was the only core variable that has been repeatedly validated and widely adopted by most investigators. As a commonly used laboratory indicator, D-dimer is widely applied in clinical practice as a biomarker related to VTE risk and is frequently used in early screening and risk assessment (47). Elevated D-dimer levels usually indicate active coagulation and fibrinolytic processes in the body, which may be associated with post-stroke stress responses, endothelial dysfunction, and a hypercoagulable state, thereby reflecting an underlying tendency toward thrombosis (48).

In addition to D-dimer, age was included in only two of the models analyzed in this study, however, a large body of previous research has consistently identified age as an important risk factor for both stroke and VTE (49, 50). With declining mobility in the elderly, venous blood flow velocity in the lower limbs decreases, accompanied by gradual increases in thrombotic factors, von Willebrand factor, and plasminogen activator inhibitor-1 in vascular endothelial cells, while the levels of antithrombotic factors (such as antithrombin III and heparin) decrease accordingly. This imbalance between coagulation and anticoagulation markedly increases the risk of thrombosis (51). Other predictors were reported only in individual models, indicating considerable variability in variable selection across existing studies and a lack of consistency in selection criteria and methodology, which may limit model comparability and contribute to instability in predictive performance. These findings underscore the need for more standardized and transparent approaches to predictor selection, with clearer clinical and methodological justification in future model development.

### 4.4 Study limitations

This study also has several limitations. To begin with, the review was restricted to publications in Chinese and English, which may have led to the omission of potentially relevant studies published in other languages. Another important limitation is that all of the included models were judged to have a high risk of bias, which restricts the reliability and generalizability of their reported performance. In addition, the apparent performance reported in the included studies may be subject to optimism bias, as most models were developed and evaluated within the same datasets without independent external validation. Furthermore, most of the data sources were derived from Chinese patient populations, which may limit the applicability of the findings to other demographic or geographic settings. Lastly, only seven studies met the eligibility criteria, which constrained the possibility of conducting adequately powered subgroup or meta-regression analyses to further investigate sources of heterogeneity.

## 5 Conclusions

This systematic review identified seven VTE prediction models developed for stroke patients. Reported discrimination ranged from 0.781 to 0.978, suggesting moderate to high apparent performance. However, all models were judged to be at high overall risk of bias according to the PROBAST tool. The main problems included insufficient outcome events, inadequate handling of missing data, lack of justification for variable transformations, and the absence of external validation, among others. These limitations may compromise the reliability of the models, restricting their clinical applicability in the assessment of VTE risk in stroke patients. Therefore, future studies should prioritize multicenter, large-scale, and prospective cohort studies to overcome these shortcomings. Adherence to standardized development frameworks, such as the PROBAST checklist and the TRIPOD statement, is essential to minimize bias and improve the quality of stroke VTE risk prediction models. In clinical practice, prediction models should also be continuously optimized and recalibrated to enhance their accuracy and reduce the incidence of adverse outcomes in stroke patients, ultimately ensuring the safety and effectiveness of clinical decision-making.

## Data Availability

The original contributions presented in the study are included in the article/Supplementary Material, further inquiries can be directed to the corresponding author.
