# Supplementary material for: Risk prediction models for venous thromboembolism in stroke: a systematic review and meta-analysis
Source: Front Cardiovasc Med. 2026 Apr 23;13:1788144. doi: 10.3389/fcvm.2026.1788144 (PMC13149398; doi:10.3389/fcvm.2026.1788144)
Supplement: Supplementary file 1 [file Table1.docx]

Table 1. Search strategy for the PubMed database

| No. | Search strategy |
| --- | --- |
| #1 | "Stroke"[Mesh] |
| #2 | ((((((((((((((((((((((((((Stroke, Acute[Title/Abstract]) OR (Acute Stroke[Title/Abstract])) OR (Acute Strokes[Title/Abstract])) OR (Strokes, Acute[Title/Abstract])) OR (Cerebrovascular Accident[Title/Abstract])) OR (Cerebrovascular Accidents[Title/Abstract])) OR (Cerebral Stroke[Title/Abstract])) OR (Cerebral Strokes[Title/Abstract])) OR (Stroke, Cerebral[Title/Abstract])) OR (Strokes, Cerebral[Title/Abstract])) OR (Cerebrovascular Apoplexy[Title/Abstract])) OR (Apoplexy, Cerebrovascular[Title/Abstract])) OR (Vascular Accident, Brain[Title/Abstract])) OR (Brain Vascular Accident[Title/Abstract])) OR (Brain Vascular Accidents[Title/Abstract])) OR (Vascular Accidents, Brain[Title/Abstract])) OR (Cerebrovascular Stroke[Title/Abstract])) OR (Cerebrovascular Strokes[Title/Abstract])) OR (Stroke, Cerebrovascular[Title/Abstract])) OR (Strokes, Cerebrovascular[Title/Abstract])) OR (Apoplexy[Title/Abstract])) OR (CVA (Cerebrovascular Accident[Title/Abstract]))) OR (CVAs (Cerebrovascular Accident[Title/Abstract]))) OR (Cerebrovascular Accident, Acute[Title/Abstract])) OR (Acute Cerebrovascular Accident[Title/Abstract])) OR (Acute Cerebrovascular Accidents[Title/Abstract])) OR (Cerebrovascular Accidents, Acute[Title/Abstract]) |
| #3 | #1 OR #2 |
| #4 | (("Venous Thromboembolism"[Mesh])) OR "Venous Thrombosis"[Mesh] |
| #5 | ((((((((((((((((((((((((Thrombosis, Venous[Title/Abstract]) OR (Thromboses, Venous[Title/Abstract])) OR (Venous Thromboses[Title/Abstract])) OR (Phlebothrombosis[Title/Abstract])) OR (Phlebothromboses[Title/Abstract])) OR (Deep Vein Thrombosis[Title/Abstract])) OR (Deep Vein Thromboses[Title/Abstract])) OR (Thromboses, Deep Vein[Title/Abstract])) OR (Vein Thromboses, Deep[Title/Abstract])) OR (Vein Thrombosis, Deep[Title/Abstract])) OR (Thrombosis, Deep Vein[Title/Abstract])) OR (Deep Venous Thrombosis[Title/Abstract])) OR (Deep Venous Thromboses[Title/Abstract])) OR (Thromboses, Deep Venous[Title/Abstract])) OR (Thrombosis, Deep Venous[Title/Abstract])) OR (Venous Thromboses, Deep[Title/Abstract])) OR (Venous Thrombosis, Deep[Title/Abstract])) OR (Deep-Vein Thrombosis[Title/Abstract])) OR (Deep-Vein Thromboses[Title/Abstract])) OR (Thromboses, Deep-Vein[Title/Abstract])) OR (Thrombosis, Deep-Vein[Title/Abstract])) OR (Deep-Venous Thrombosis[Title/Abstract])) OR (Deep-Venous Thromboses[Title/Abstract])) OR (Thromboses, Deep-Venous[Title/Abstract])) OR (Thrombosis, Deep-Venous[Title/Abstract]) |
| #6 | #4 OR #5 |
| #7 | ((((((((("Risk Factors"[Mesh]) OR "Risk Assessment"[Mesh]) OR "Early Warning Score"[Mesh]) OR "Nomograms"[Mesh])) OR "Logistic Models"[Mesh]) OR "Models, Statistical"[Mesh]) OR "Support Vector Machine"[Mesh]) OR "Decision Trees"[Mesh]) OR "Decision Support Techniques"[Mesh] |
| #8 | ((((((((((((((((((((((((((((Predict*[Title/Abstract]) OR (Prognose*[Title/Abstract])) OR (Warning*[Title/Abstract])) OR (Model*[Title/Abstract])) OR (Risk Instrument[Title/Abstract])) OR (Risk Score[Title/Abstract])) OR (Risk Scoring[Title/Abstract])) OR (Risk Index[Title/Abstract])) OR (Risk Prediction Model[Title/Abstract])) OR (Risk Assessment Instrument[Title/Abstract])) OR (Risk Assessment Tool[Title/Abstract])) OR (Risk Assessment Acore[Title/Abstract])) OR (Risk Assessment Measure[Title/Abstract])) OR (Risk Evaluation[Title/Abstract])) OR (Risk Factors[Title/Abstract])) OR (Risk Appraisal[Title/Abstract])) OR (Risk Stratification[Title/Abstract])) OR (Predictors[Title/Abstract])) OR (Risk-Ttratified[Title/Abstract])) OR (Hierarchical Risk[Title/Abstract])) OR (Nomogram*[Title/Abstract])) OR (Neural Network[Title/Abstract])) OR (Neural Networks[Title/Abstract])) OR (Support Vector Machine[Title/Abstract])) OR (Support Vector Machines[Title/Abstract])) OR (Decision Tree[Title/Abstract])) OR (Decision Trees[Title/Abstract])) OR (Machine Learning[Title/Abstract])) OR (Machine Learnings[Title/Abstract]) |
| #9 | #7 OR #8 |
| #10 | #3 AND #6 AND #9 |
